# JP4-039 Mitigates Cisplatin-Induced Acute Kidney Injury by Inhibiting Oxidative Stress and Blocking Apoptosis and Ferroptosis in Mice

**DOI:** 10.3390/antiox13121534

**Published:** 2024-12-15

**Authors:** Merlin Airik, Kacian Clayton, Peter Wipf, Rannar Airik

**Affiliations:** 1 Division of Nephrology, Department of Pediatrics, UPMC Children’s Hospital of Pittsburgh, Pittsburgh, PA 15224, USA; 2 Department of Chemistry, University of Pittsburgh, Pittsburgh, PA 15260, USA; 3 Department of Cell Biology, University of Pittsburgh, Pittsburgh, PA 15260, USA

**Keywords:** acute kidney injury, oxidative stress, antioxidants, JP4-039, apoptosis, ferroptosis

## Abstract

Cisplatin is a commonly used chemotherapeutic agent in the treatment of a wide array of cancers. Due to its active transport into the kidney proximal tubule cells, cisplatin treatment can cause a buildup of this nephrotoxic compound in the kidney, resulting in acute kidney injury (AKI). About 30% of patients receiving cisplatin chemotherapy develop cisplatin-induced AKI. JP4-039 is a mitochondria-targeted reactive oxygen species (ROS) and electron scavenger. Recent studies have shown that JP4-039 mitigates a variety of genotoxic insults in preclinical studies in rodents by suppressing oxidative stress-mediated tissue damage and blocking apoptosis and ferroptosis. However, the benefits of JP4-039 treatment have not been tested in the setting of AKI. In this study, we investigated the potential renoprotective effect of JP4-039 on cisplatin-induced AKI. To address this goal, we treated mice with JP4-039 before or after cisplatin administration and analyzed them for functional and molecular changes in the kidney. JP4-039 co-administration attenuated cisplatin-induced renal dysfunction and histopathological changes. Upregulation of tubular injury markers was also suppressed by JP4-039. Mechanistically, JP4-039 suppressed lipid peroxidation, prevented tissue oxidative stress, and preserved the glutathione levels in cisplatin-injected mice. An increase in cisplatin-induced apoptosis and ferroptosis was also alleviated by the compound. Moreover, JP4-039 inhibited cytokine overproduction in cisplatin-injected mice. Together, our findings demonstrate that JP4-039 is a promising therapeutic agent against cisplatin-induced kidney injury.

## 1. Introduction

Cisplatin is a commonly used highly effective therapeutic in cancer treatment [1]. However, its use is limited by its nephrotoxicity, which occurs in about 30% of patients receiving cisplatin chemotherapy [2,3]. One of the mechanisms of cisplatin nephrotoxicity is the induction of reactive oxygen species (ROS) due to disruptions in the mitochondrial respiratory chain [4]. ROS, such as superoxide radical anions and hydroxyl radicals, are highly reactive molecules that can damage cellular components, including lipids in cell membranes by allylic peroxidation [5]; increase mitochondrial membrane permeability, and induce caspase-3 activity, resulting in tubular cell apoptotic death [6], and ferroptosis [7,8], another form of cell death which has been recently implicated in AKI pathophysiology [8,9,10]. Cisplatin also affects the cellular redox balance by binding to and depleting free glutathione (GSH) levels in the cell [2,4]. GSH is a major antioxidant molecule, whose production is regulated by the transcriptional activity of the nuclear factor erythroid 2-related factor 2 (Nrf2) [11]. GSH is also necessary for the function of glutathione peroxidase 4 (Gpx4) [12], an antioxidant enzyme whose activity has been recently demonstrated to be critical for protecting kidney proximal tubular cells from lipid peroxidation and ferroptosis [9,13].

There are currently no approved therapeutic agents to treat AKI. Since cisplatin-induced AKI remains a major unmet medical need without any pharmacological interventions, we decided to explore the nephroprotective effects of a novel mitochondria-targeted ROS and electron scavenger, JP4-039 [14], in a cisplatin AKI model. JP4-039 is a potent antioxidant that contains a hemigramicidin S tail, which facilitates its accumulation in mitochondrial membranes and a 4-amino-TEMPO nitroxide headgroup, which functions as a ROS scavenger [15] and exerts dismutase and catalase activities. JP4-039 has demonstrated protective effects in several preclinical models, mitigating tissue damage, preserving antioxidant reserves, and maintaining cellular integrity under stress conditions such as radiation exposure [16] or toxic insults [17]. Previously, we have shown that JP4-039 successfully halted the progression of kidney disease in a genetic model of chronic kidney disease [18]. In addition, JP4-039 has been demonstrated to inhibit both apoptotic and ferroptotic cell death in various in vivo and in vitro disease models [17,18,19], but whether JP4-039 prevents kidney tubular cell death after injury has not been investigated to date. Furthermore, whether JP4-039 protects wild type mice from acute kidney injury after cisplatin injury is not known. Thus, in the current study, we examined the effect of JP4-039 on cisplatin-induced kidney injury and explored the mechanism.

## 2. Materials and Methods

### 2.1. Mouse Lines Used and Study Approval

Eight-to-ten-week-old male mice (129Sv-Elite, Charles River, Wilmington, MA, USA) were used in this study. All mice were maintained under SPF conditions in an ambient temperature of 20–22 degrees, a humidity of 50–70%, and a 12/12 h light/dark cycle. All animal experiments were reviewed and approved by the Institutional Animal Care and Use Committee of the University of Pittsburgh and were performed in accordance with the institutional guidelines. Animals were randomly assigned to 4 experimental groups, each consisting of 5 male mice. Group 1 (hereafter, ctrl) is a vehicle control group, in which mice were injected with normal saline (0.9% NaCl, a vehicle for cisplatin) and 10 mL of DMSO (vehicle for JP4-039); group 2 (hereafter, C) received a single intraperitoneal (i.p.) dose of cisplatin (10 mg/kg); group 3 (hereafter, C + JP4-039) received a single dose of cisplatin (10 mg/kg) followed by gavage feeding of JP4-039 (20 mg/kg) 24 h later; and group 4 (hereafter, JP4-039 + C) were gavage-fed with JP4-039 (20 mg/kg) followed by cisplatin (10 mg/kg) 1 h later. JP4-039 was synthesized and QC’d in the laboratory of Dr. P. Wipf and stored at −80 °C as a stock solution of 0.1 mg/mL in DMSO. JP4-039 stock solution was dissolved in 50% PEG-400/50% H_2_O on the day of gavage feeding. Mice were euthanized 72 h (day 3) after cisplatin injection to harvest blood and kidneys.

### 2.2. Measurement of Renal Function

Renal function was assessed with blood urea nitrogen (BUN). Serum was separated from red blood cells by centrifugation at 2000× *g* for 15 min at 4 °C. BUN levels in serum were measured using a kit per the manufacturer’s protocol (#K024-H1, Cayman Chemical, Ann Arbor, MI, USA).

### 2.3. Histological Analysis of Kidney

Mouse kidneys were fixed with 4% paraformaldehyde overnight at 4 °C, embedded in paraffin, and sectioned at 4 μm thickness. Staining with periodic acid–Schiff (PAS; #395B-1KT Sigma-Aldrich, St. Louis, MO, USA) reagents was performed according to the manufacturer’s protocol. Images were captured using a light microscope (Leica, Wetzlar, Germany).

### 2.4. Immunofluorescence Staining (IF)

For immunofluorescence staining, paraffin-embedded sections were sequentially treated through deparaffinization, hydration, and antigen retrieval via incubation with citrate buffer (pH6). To reduce nonspecific binding, the sections were blocked in 10% donkey serum, 0.5% Triton, and 1% BSA in PBS for 1 h at RT and incubated with primary antibodies overnight at 4 °C. Following overnight incubation, the slides were incubated with a secondary antibody for 1 h and 30 min, rinsed with PBS, and counterstained with DAPI. Images were obtained using a Leica SP8 confocal microscope. Fluorescence was quantified using the ImageJ software version 2.14.0/1.54f (NIH, Bethesda, MD, USA).

### 2.5. Immunohistochemistry Staining (IHC)

For immunohistochemical studies, a standard manufactory protocol was followed. After the deparaffinization procedures, antigen retrieval was performed by using citrate buffer (pH6). Endogenous peroxidases were inactivated using 3% hydrogen peroxide, followed by blocking with 10% donkey serum, 0.5% Triton, and 1% BSA in PBS for 1 h at room temperature. Sections were incubated with primary antibodies overnight at 4 °C. The next day, the sections were washed and incubated for 1 h with a secondary antibody. Histochemical reactions were detected with the ABC Elite kit (Vector Laboratories, Newark, CA, USA) according to the manufacturer’s instructions. Images were captured using a light microscope (Leica, Wetzlar, Germany).

### 2.6. Antibodies

Primary antibodies and lectins used in this study are shown in Supplementary Table S1. Secondary antibodies included donkey anti-rabbit Alexa Fluor 594, donkey anti-rabbit Alexa Fluor 488, donkey anti-mouse Alexa Fluor 594, or donkey anti-mouse Alexa Fluor 488 (all from Molecular Probes, Eugene, OR, USA), as well as goat anti-rabbit IgG-HRP (sc-2004, Santa Cruz, Dallas, TX, USA). Samples were mounted in Fluoromount Aqueous Mounting Medium (F4680, Sigma-Aldrich, St. Louis, MO, USA).

### 2.7. RNA Extraction and Real-Time PCR

RNA was isolated from mouse kidneys using a Quick-RNA Miniprep kit (R1055, Zymo Research, Irvine, CA, USA), and complementary DNA was synthesized from 500 ng of total RNA using an iScript cDNA synthesis kit (Bio-Rad, catalog no.1708890, Hercules, CA, USA) according to the manufacturer’s instructions. Real-time PCR analysis was performed with 2× SYBR Green Master Mix (Bio-Rad, catalog no. 1725270, Hercules, CA, USA) and run on a CFX96 real-time PCR system (Bio-Rad, Hercules, CA, USA). Relative expression levels of mRNAs were normalized to Gapdh or 18SrRNA. qPCR primers used in this study are shown in Supplementary Table S2.

### 2.8. Western Blotting

Kidney tissues were lysed with RIPA lysis buffer containing protease and phosphate inhibitors and homogenized with a sonicator. Lysates were clarified via centrifugation at 16,000× *g* for 30 min at 4 °C and protein concentration was measured using a DC protein assay kit (#500-0116, Bio-Rad). Gel electrophoresis of equal amount of tissue lysates was performed using the Bolt system (Invitrogen, Waltham, MA, USA) and transferred onto the PVDF membrane. Membranes were blocked with 5% nonfat milk in TBST and probed with primary and secondary antibodies, respectively. The immunoblots were visualized with ECL reagents according to the manufacturer’s instructions. Protein bands were normalized with Gapdh.

### 2.9. Measurement of Cellular ROS

ROS generation in kidneys was assayed using CM-H2DCFDA (C6827, Invitrogen, Waltham, MA, USA). Unfixed kidney cryosections were incubated with 5 μM CM-H2DCFDA in PBS for 30 min at 37 °C in a light-protected humidified chamber. Samples were then washed three times, and images were obtained with a Leica SP8confocal microscope. The intensity of the fluorescence was quantified by the NIH Image J software.

### 2.10. Glutathione Assay

Kidneys were lysed in RIPA lysis buffer and samples were then assayed for total glutathione (GSH) content according to the manufacturer’s instructions (Abcam, ab205811, Cambridge, UK). The protein levels were determined using the DC Protein Assay Kit (Bio-Rad). Values were normalized to the protein content of whole homogenates.

### 2.11. Quantification

The area occupied by Kim1-, Collagen1a1-, Fibronectin1-, CM-H2DCD-, and 4-HNE-stained sections was evaluated using ImageJ software [20]. For quantification, 6 randomly selected, non-overlapping fields were imaged at 400× magnification in the renal cortex. The values obtained were expressed as a percentage of the whole cortical area.

### 2.12. Statistical Methods

Statistical analysis was performed using GraphPad Prism 10, version 10.4.1 (532) (GraphPad software, Boston, MA, USA). Statistical tests are two-tailed, unpaired Student’s *t*-tests or an ordinary one-way or two-way ANOVA followed by Tukey’s post hoc test for multiple group comparisons. All results are reported as means ± SEM. Significance was determined at *p* < 0.05 and represented by * to denote *p* < 0.05, ** *p* < 0.01, *** *p* < 0.001, and **** *p* < 0.0001.

## 3. Results

### 3.1. JP4-039 Protects Against Cisplatin-Induced Kidney Injury

**Figure 1:**
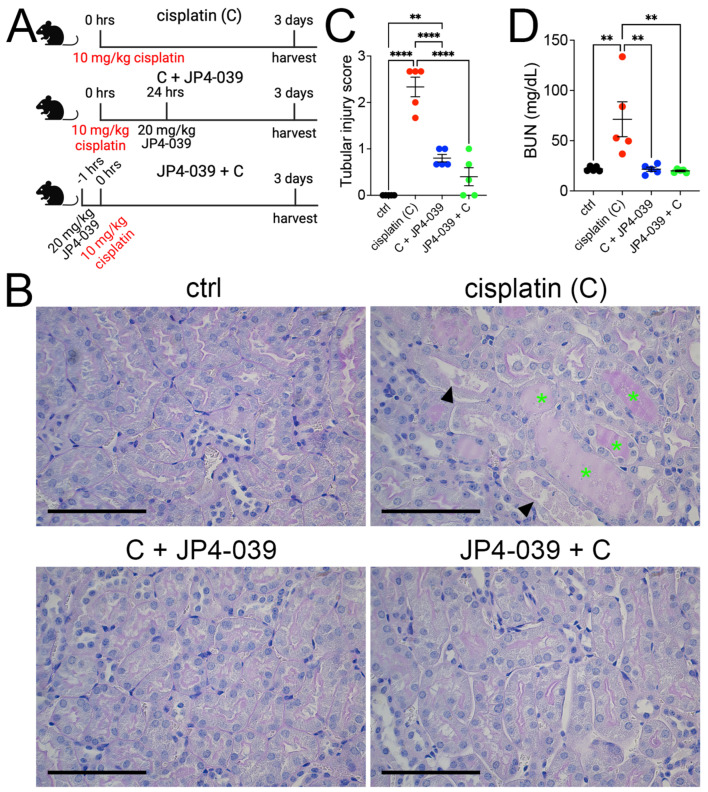
Cisplatin-induced kidney injury and loss of function is mitigated by JP4-039. (**A**) Overview of the cisplatin AKI and JP4-039 administration protocols. Cisplatin was administered to three cohorts of 129Sv-Elite mice; cisplatin cohort (**C**) received a single dose of cisplatin (10 mg/kg), cohort 2 (C + JP4-039) received a single dose of cisplatin (10 mg/kg), followed by JP4-039 (20 mg/kg) 24 h later, and cohort 3 (JP4-039 + C) was injected with JP4-039 (20 mg/kg) followed by cisplatin (10 mg/kg) 1 h later. Control mice (ctrl) were injected with normal saline and DMSO (vehicles for cisplatin and JP4-039, respectively). Kidneys and blood were collected for analysis 3 days after cisplatin injection. (**B**) Histological analysis of kidney sections via periodic acid–Schiff (PAS) staining reveals that JP4-039 treatment preserves the kidneys from cisplatin injury. Green asterisks, protein casts; arrowheads, loss of brush-border. Scale bars: 100 μm. (**C**) Blood urea nitrogen (BUN) measurements demonstrate AKI in cisplatin-only but not in control and JP4-039-treated mice. Ordinary one-way ANOVA with Tukey’s multiple comparison ** *p* < 0.01, **** *p* < 0.0001, *n* = 5 each. (**D**) Tubular injury scores based on PAS-stained kidney sections. Ordinary one-way ANOVA with Tukey’s multiple comparison ** *p* < 0.01, *n* = 5 each. (**C**,**D**) Data are presented as the mean ± SEM. A two-way ANOVA with Tukey’s post hoc analysis.

To investigate whether JP4-039 administration can modulate cisplatin AKI in wild type mice, we used a low-dose cisplatin (10 mg/kg) kidney injury model in 129Sv-Elite mice. Cisplatin (C) was injected intraperitoneally into three cohorts of mice (Figure 1A). Control mice (ctrl) were administered an equal amount of vehicle solution. JP4-039 was administered by gavage feeding either 24 h after cisplatin injection (C + JP4-039) to assess for its therapeutic activity or 1 h before cisplatin (JP4-039 + C) to investigate its efficacy in preventing cisplatin nephrotoxicity (Figure 1A). Kidney histology and blood chemistry were analyzed 72 h after cisplatin injection. As expected, cisplatin administration caused the mice to develop histological changes associated with AKI, such as loss of proximal tubule brush border, tubular damage, cast formation, and basement membrane thickening when compared to untreated kidneys. In contrast, these pathologic changes were markedly alleviated in mice who were co-treated with JP4-039, demonstrating a prevention of AKI (Figure 1B,C). In line with the histologic data, serum BUN levels were significantly increased in cisplatin-injected wild-type mice when compared to JP4-039-treated mice (Figure 1D). Taken together, these results indicate that JP4-039 mitigates cisplatin-induced kidney injury in mice, both when administered before or after cisplatin exposure.

### 3.2. JP4-039 Blocks the Induction of Kidney Tubular Injury in Cisplatin Exposed Kidneys

**Figure 2:**
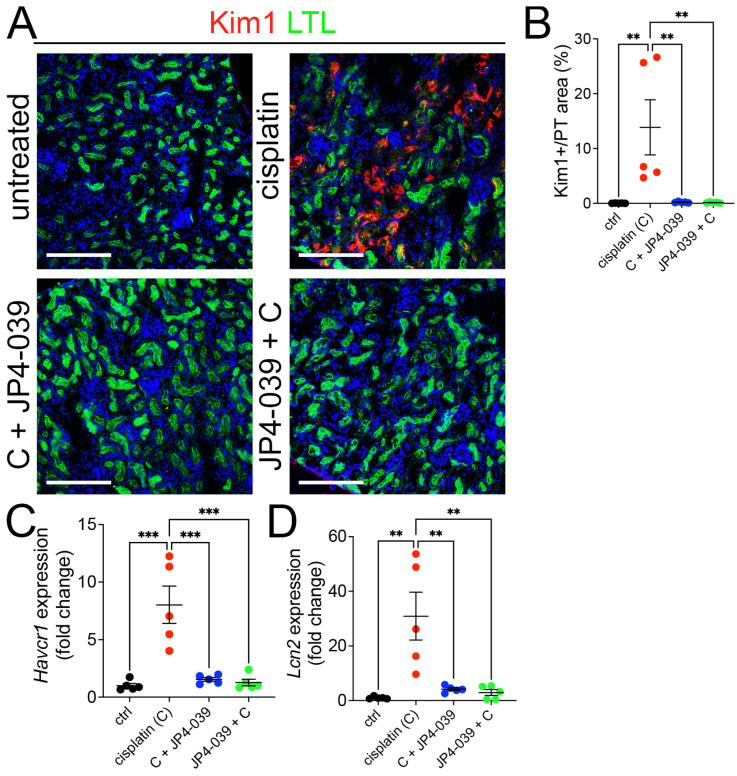
JP4-039 administration mitigated tubular injury in cisplatin AKI. (**A**) Representative images of Kim1 immunofluorescence staining in control (untreated) and cisplatin ± JP4-039-treated kidneys on day 3 after AKI induction, Kim1 (red), LTL (green), DAPI (blue). Scale bar: 200 μm. (**B**) Quantification of KIM1-positive area in LTL-positive proximal tubules shows that prophylactic and therapeutic JP4-039 administrations suppresses KIM1 expression in cisplatin-injected kidneys. One way-ANOVA, ** *p* < 0.01, *n* = 5 each. (**C**) *Havcr1* expression was increased in cisplatin AKI kidneys but remained at baseline in mice co-administered with JP4-039. One way-ANOVA, *** *p* < 0.001, *n* = 5 each. (**D**) *Ngal* expression was increased in cisplatin AKI kidneys but remained at baseline in the kidneys of mice co-administered JP4-039. One way-ANOVA, ** *p* < 0.01, *n* = 5 each. (**B**–**D**) Data are presented as the mean ± SEM. One way-ANOVA with Tukey’s post hoc analysis.

To determine whether JP4-039 mitigates AKI by suppressing tubular cell injury in cisplatin-treated mice, we co-stained kidney sections with antibodies against the kidney tubular injury marker, i.e., kidney injury molecule-1 (Kim1) and the proximal tubule marker *Lotus tetragonolobus* lectin (LTL). While cisplatin administration induces a strong Kim1 expression in the kidneys by 72 h, prophylactic and therapeutic treatments with JP4-039 blocked Kim1 expression (Figure 2A,B). Quantitative RT-PCR analysis confirmed that the expression of the kidney injury markers—*hepatitis a virus cellular receptor 1* (*Havcr1*, encodes Kim1) and *lipocalin 2* (*Lcn2*)—remained at baseline in JP4-039 and cisplatin co-treated mice, compared to cisplatin-administered mice (Figure 2C,D). Together, these data show that JP4-039 therapy is equally effective at protecting tubular cells from cisplatin injury when administered either before or after cisplatin administration.

### 3.3. Reduced Tubular Interstitial Fibrogenesis in Mice Treated with JP4-039

**Figure 3:**
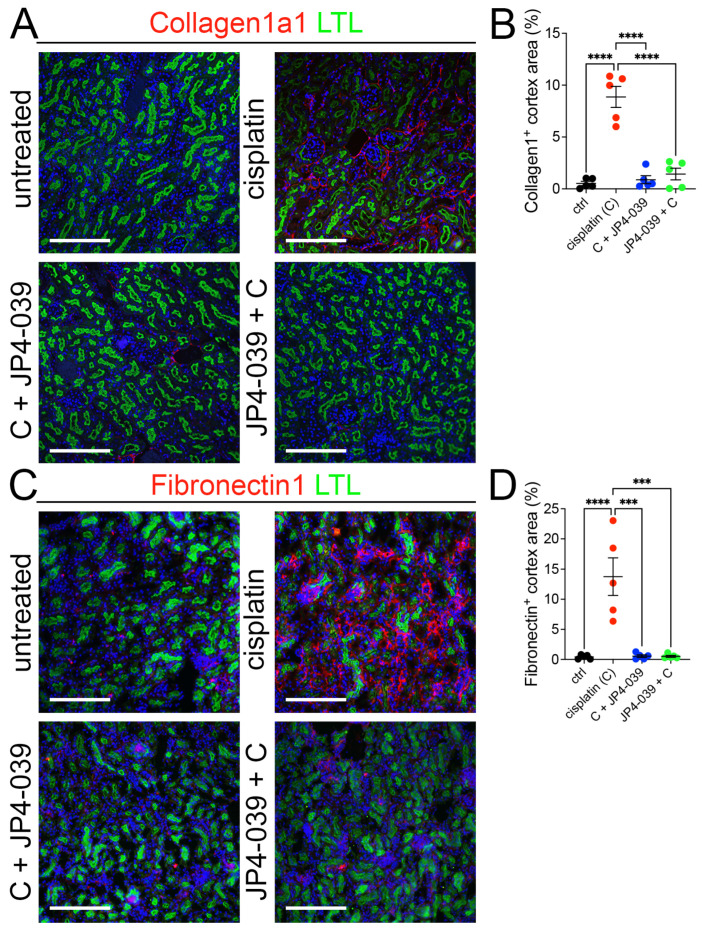
JP4-039 blocked the development of tubular interstitial fibrosis in cisplatin AKI. (**A**) Representative images of Collagen1 immunofluorescence staining in control (untreated) and cisplatin ± JP4-039-treated kidneys on day 3 after AKI induction; collagen 1a1 (red), LTL (green), and DAPI (blue). Scale bar: 200 μm. (**B**) Quantification of collagen 1-positive area in the cortical region shows that prophylactic and therapeutic JP4-039 administrations suppress Col1a expression in cisplatin-injected kidneys. One way-ANOVA, **** *p* < 0.0001, *n* = 5 each. (**C**) Representative images of fibronectin 1 immunofluorescence staining in control (untreated) and cisplatin ± JP4-039-treated kidneys on day 3 after AKI induction; fibronectin 1 (red), LTL (green), DAPI (blue). Scale bar: 200 μm. (**D**) Quantification of fibronectin 1-positive area in the cortical region shows that prophylactic and therapeutic JP4-039 treatments suppress Fn1 expression in cisplatin-injected kidneys. (**B**,**D**) Data are presented as the mean ± SEM. One way-ANOVA, *** *p* < 0.001, **** *p* < 0.0001, *n* = 5 each.

Interstitial fibrosis develops downstream of tubular injury in AKI. To examine whether JP4-039-treated kidneys have reduced fibrosis we examined the expression of two fibrosis markers—collagen 1a1 and fibronectin 1—in kidney sections. Both fibrosis-associated proteins were expressed at significantly higher levels in cisplatin-injured mice when compared to untreated or JP4-039-treated mice (Figure 3A–D). These results demonstrate that JP4-039 administration prevents the development of interstitial fibrosis in cisplatin injured kidneys.

### 3.4. JP4-039 Attenuates the Expression of Inflammatory Cytokines and Chemokines in Cisplatin Injured Kidneys

**Figure 4:**
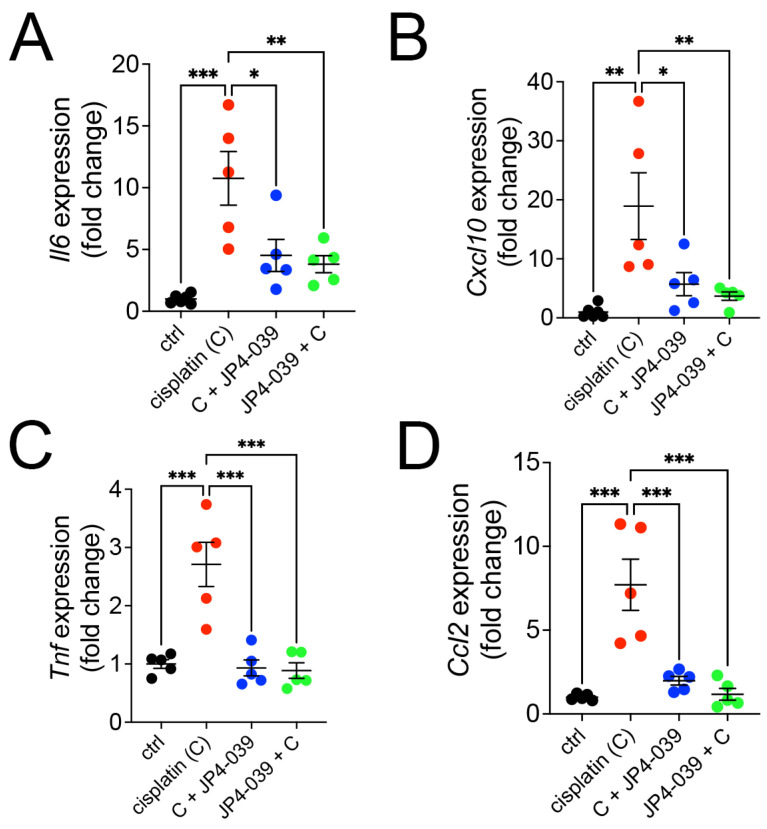
JP4-039 treatment reduced intrarenal infiltration of inflammatory cells triggered by tubular cell death. (**A**–**D**) Evaluation of the expression of several inflammatory genes—*Il6* (**A**), *Cxcl10* (**B**), *Tnf* (**C**), and *Ccl2* (**D**) by qPCR reveals a robust inflammatory signaling in cisplatin AKI, which is suppressed in mice co-treated with JP4-039. (**A**–**D**) Data are presented as the mean ± SEM. One way-ANOVA with Tukey’s post hoc analysis, * *p* < 0.05, ** *p* < 0.01, *** *p* < 0.001, *n* = 5 per group.

The inflammatory response is one of the major causes of cisplatin-induced acute kidney injury. To investigate the effect of JP4-039 on the inflammatory response in mouse renal tubular cells, we examined the expression of proinflammatory cytokines, including *Il6* and *Tnf*, in each group. Analysis by quantitative RT-PCR showed that while cisplatin treatment significantly induced the expression of *Il6*, *Cxcl10*, *Tnf*, and *Ccl2* in wild-type kidneys, cotreatment with JP4-039 attenuated the increase in cisplatin-induced inflammatory factors (Figure 4A–D). Together, these data demonstrated that JP4-039 therapy significantly attenuates the cisplatin-induced inflammatory response.

### 3.5. JP4-039 Blocks Oxidative Stress in the Cisplatin Injury

**Figure 5:**
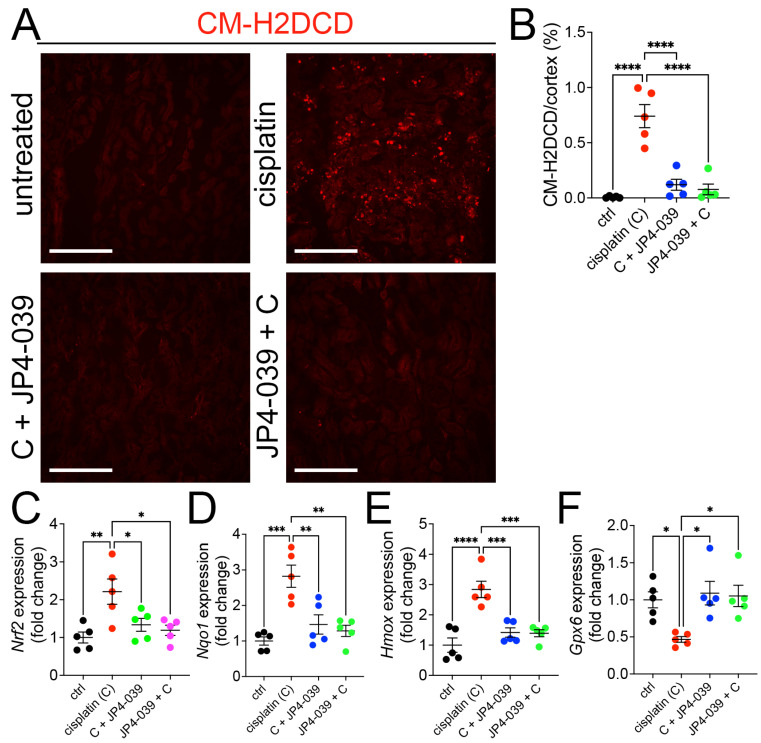
JP4-039 blocked the generation of reactive oxygen species (ROS) and activation of the Nrf2 antioxidant pathway in cisplatin-treated kidneys. (**A**) CM-H2DCFDA staining in untreated and cisplatin ± JP4-039-treated kidneys. Scale bar: 200 μm. (**B**) Quantification of the CM-H2DCFDA. **** *p* < 0.0001, n = 5. (**C**–**F**) Expression of *Nrf2* (**C**) and its target genes, *Nqo1* (**D**) and *Hmox* (**E**), was increased in cisplatin AKI kidneys but remained at baseline in mice co-administered with JP4-039, while *Gpx6* (**F**) expression was reduced in AKI kidneys but preserved at baseline in JP4-039 administered kidneys. One way-ANOVA, * *p* < 0.05, ** *p* < 0.01, *** *p* < 0.001, **** *p* < 0.0001, *n* = 5 each. (**B**–**F**) Data are presented as the mean ± SEM. One way-ANOVA with Tukey’s post hoc analysis.

Oxidative stress has been reported to be the main mechanism for cisplatin-induced apoptosis. To investigate the level of intracellular ROS, we used the sensitive fluorescent probe CM-H2DCFDA, which labels total cellular ROS in unfixed tissue sections. We observed a significant increase in intracellular ROS levels in cisplatin-treated kidneys compared to untreated kidneys. However, treatment with JP4-039 significantly reduced CM-H2DCFDA signal intensity in cisplatin-treated kidneys demonstrating functional protection from ROS (Figure 5A,B). Oxidative stress induces Nrf2 expression in the kidney [21,22]. Nrf2 is a master regulator if genes are involved in antioxidative response [22]. To confirm that JP4-039 protects the kidneys from cisplatin injury by blocking oxidative stress, we examined the expression of *Nrf2* and its target genes (*Nqo1*, *Hmox1*, *Gpx6*) in the kidneys. Nrf2 and its target genes’ expression remained at baseline in the kidneys of JP4-039-treated mice, comparable with uninjured wild type kidneys, indicating that JP4-039 blocked the formation of oxidative stressors after cisplatin treatment (Figure 5C–F). However, mice which were treated with cisplatin only showed significantly elevated expression of Nrf2 signaling pathway genes, indicating severe oxidative stress (Figure 5C–E). Interestingly, the expression of *Gpx6* was downregulated in cisplatin-injured kidneys, suggesting a Nrf2-independent regulation of *Gpx6* in cisplatin AKI (Figure 5F). Together, these results show that JP4-039 blocks the accumulation of cellular ROS in cisplatin-treated kidneys and subsequently prevents the activation of the Nrf2 pathway.

### 3.6. JP4-039 Ameliorates Cisplatin-Induced Tubular Death by Inhibiting Apoptosis and Ferroptosis

**Figure 6:**
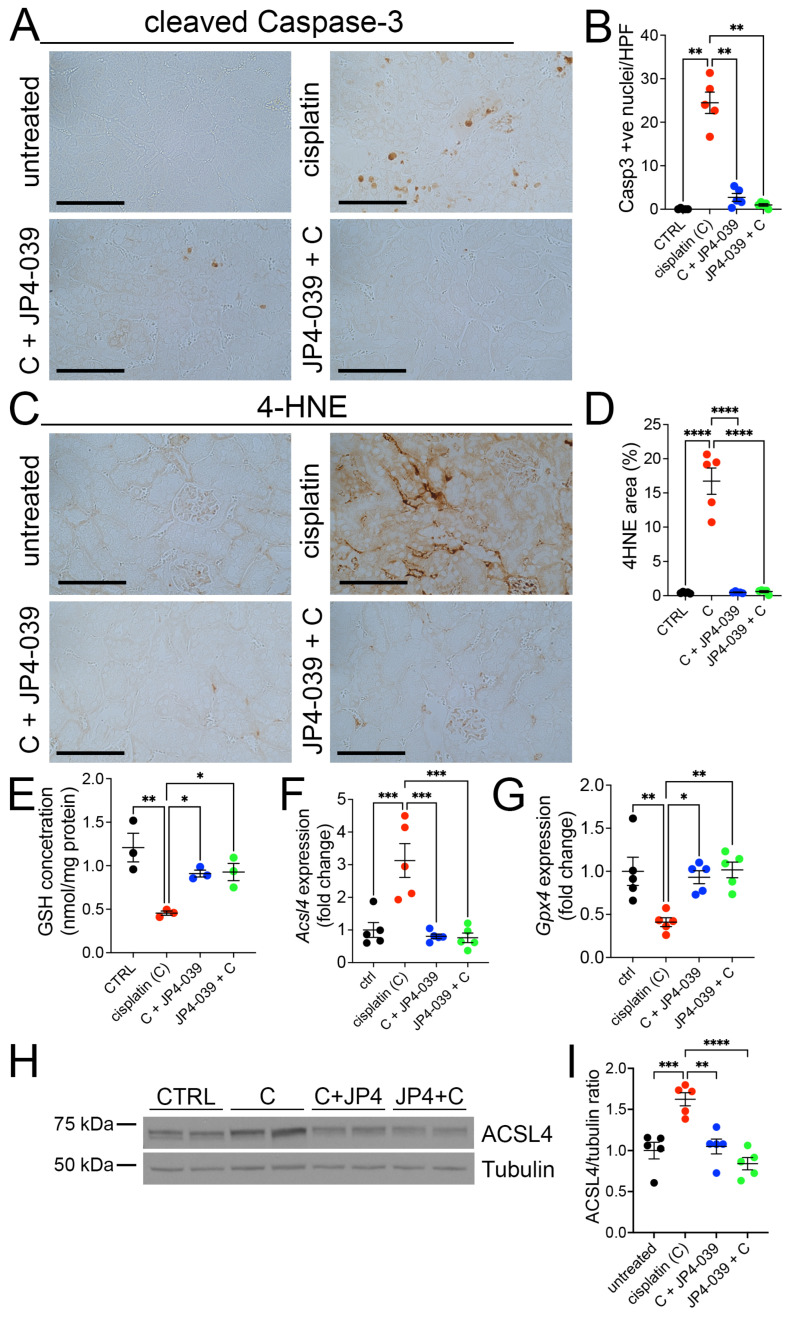
JP4-039 reduced apoptosis and ferroptosis in tubular epithelial cells in cisplatin-injured kidneys. (**A**) Representative images of cleaved Caspase-3 immunohistochemistry staining in untreated and cisplatin ± JP4-039-treated kidneys. Scale bar: 100 μm. (**B**) Quantification of cleaved Caspase-3 positive cells in the kidney cortex. ** *p* < 0.01, *n* = 5 per group. (**C**) 4-Hydroxynonenal (4-HNE) staining in control and AKI ± JP4-039 kidneys showed that JP4-039 mitigated the cisplatin-induced accumulation of lipid peroxidation in kidneys 3 days after cisplatin administration. Scale bar: 100 μm. (**D**) Quantification of 4HNE positive area in the kidney cortical area. **** *p* <0.0001, *n* = 5 per group. (**E**) Quantification of free GSH concentration in whole kidney lysates. * *p* < 0.05, ** *p* < 0.01, *n* = 3 per group. (**F**,**G**) qPCR analysis of *Acsl4* and *Gpx4* expression in kidneys normalized to *Gapdh,* * *p* < 0.05, ** *p* < 0.01, *** *p* < 0.001, *n* = 5 per group. (**H**,**I**) Western blot and quantification of kidney Acsl4 protein. Tubulin is a loading control. ** *p* < 0.01, *** *p* < 0.001, **** *p* <0.0001, *n* = 5 per group. (**B**,**D**–**F**,**G**,**I**) Data are presented as the mean ± SEM. One way-ANOVA with Tukey’s post hoc analysis.

A major effect of cisplatin cytotoxicity is the induction of cell death [23]. Thus, we next investigated whether JP4-039 elicits its renoprotective effect by modulating cisplatin-induced cell death. Analysis of kidney sections stained with antibodies against the apoptosis marker, cleaved Caspase-3, revealed a significant increase in the number of apoptotic tubular cells in cisplatin-treated kidneys (Figure 6A), indicating that cisplatin induces kidney cell apoptosis. In contrast, cotreatment with JP4-039 prevented this increase in cleaved Caspase-3 positive cells (Figure 6A,B), indicating that JP4-039 inhibited cisplatin-induced cell death in the kidney.

In addition to the activation of pro-apoptotic signaling, cisplatin affects cell viability by depleting free glutathione (GSH) stores in the cell, which are essential for regulating cellular antioxidant defenses and critical for blocking the accumulation of lipid peroxides and induction of ferroptosis [9]. Our analysis showed that cisplatin treatment led to reduced cellular GSH levels in the kidneys; however, these alterations were prevented in mice co-administered with JP4-039 (Figure 6E). Coincident with GSH reduction, cisplatin injection resulted in increased 4-hydroxynoneal (4-HNE) levels in the kidneys, while JP4-039 inhibited the accumulation of this highly toxic compound in the cells (Figure 6C,D), further underlying the therapeutic benefits of JP4-039 in suppressing cisplatin-induced oxidative stress in the kidney. JP4-039 is a candidate ferroptosis inhibitor [14]. In order, to explore the potential anti-ferroptotic effects of JP4-039, we analyzed the gene expression changes of two key ferroptosis regulators, namely, *acyl*-*CoA* synthetase long-chain family member 4 (*Acsl4*) and glutathione peroxidase 4 (*Gpx4*). We observed a marked increase in *Acsl4* and a down-regulation in *Gpx4* expression in cisplatin-injured kidneys. However, the expression of both genes remained at baseline levels in JP4-039-treated kidneys (Figure 6F,G), consistent with the activation of ferroptosis after cisplatin injury and its inhibition by JP4-039. Further, immunoblot analysis of Acsl4 expression in cisplatin injured and JP4-039-treated kidneys corroborated the quantitative RT-PCR based studies (Figure 6H,I), demonstrating that JP4-039 protected injured kidneys from cisplatin-induced ferroptosis. In summary, these findings suggest that JP4-039 is a highly potent inhibitor of tubular epithelial cell apoptosis and ferroptosis in cisplatin injured kidneys.

## 4. Discussion

In this study, we investigate the therapeutic potential of the mitochondrially localized electron and ROS scavenger JP4-039 in mitigating cisplatin-induced kidney injury. Our results indicate that JP4-039 administration confers renoprotection both when delivered prophylactically, 1 h before cisplatin injection, or therapeutically, 24 h after cisplatin administration in mice. Mechanistically, our results demonstrate that JP4-039 elicits its therapeutic effect by (1) inhibiting ROS generation in cisplatin exposed kidneys, which results in (2) the suppression of proinflammatory and fibrogenic signaling, and (3) inhibition of apoptotic and ferroptotic cell death pathways in the kidney tubular epithelial cells.

We and other groups have previously reported the tissue protective effects of JP4-039 in various preclinical disease models, including FAN1 kidney disease [18], total body irradiation [24], radiation-induced mucositis [19] and sulfite-induced striatal cell death [17]. In the current study, we extend these findings and demonstrate that JP4-039 is a potent therapeutic against cisplatin-induced kidney injury. Our results show that JP4-039 protects the kidneys from cisplatin-induced injury by blocking ROS generation in the tubular cells, which is one of the first pathophysiologic effects of cisplatin after its uptake in the kidney [23]. By inhibiting ROS accumulation, JP4-039 prevents the induction of overt oxidative stress in cisplatin-treated kidneys and the subsequent activation of the endogenous Nrf2 antioxidant pathway, as demonstrated by the baseline expression of *Nrf2* and its target genes, *Nqo1* and *Hmox1*, in JP4-039 administered kidneys. Unexpectedly, we found that the expression of *glutathione peroxidase 6* (*Gpx6*), a putative Nrf2 target gene in the liver [25] and kidneys [26] and a biomarker of oxidative stress in the brain [27], was regulated in the opposite direction compared to other Nrf2 targets after cisplatin injury. *Gpx6* expression was significantly downregulated in cisplatin-injured kidneys, whereas its expression was maintained at normal levels in JP4-039-treated kidneys. These results suggest that while *Gpx6* expression is regulated in response to oxidative stress, *Gpx6* is not a direct transcriptional target of Nrf2 in cisplatin-induced AKI. Counterintuitively, we observed that kidneys in which the endogenous Nrf2 antioxidant signaling pathway was markedly upregulated after cisplatin administration resulted in worse AKI outcomes compared to kidneys in which Nrf2 pathway activity remained at baseline after JP4-039 treatment. Indeed, extensive evidence from several studies shows that genetic or pharmacologic activation of the Nrf2 antioxidant response leads to improved kidney injury outcomes in ischemia-reperfusion injury (IRI) [28] and aristolochic-acid-induced [29] AKI models in mice, and conversely, suppression of Nrf2 activity by chronic administration of N-acetyl-cysteine (NAC), an antioxidant, contributes to AKI to CKD progression [30]. A plausible explanation to this seeming discrepancy is that extensive ROS do not only cause redox imbalance in the cell, which leads to Nrf2 activation, but can directly damage multiple subcellular components, including DNA [31] and lipids [32], injuries which are virtually absent in JP4-039-treated kidneys, reported here and in [18]. Moreover, our results are consistent with a recent report that endogenous Nrf2 activity is not sufficient to mitigate AKI after IRI kidney injury [33]. Here, we show that this applies to cisplatin AKI and further demonstrates that inhibition of intracellular ROS by JP4-039 without engaging the Nrf2 antioxidant pathway is sufficient to block AKI pathophysiology and tubular injury. This notion is supported by the attenuated expression of tubular injury markers (KIM1/*Havcr1* and *Lcn2*), inflammatory cytokines (*Tnf*, *Cxcl10*, *Il6*, *Ccl2*), and fibrotic markers (Col1a1 and Fn1) in JP4-039-treated kidneys.

Another important finding was that JP4-039 significantly alleviated cell death in cisplatin-treated kidneys. The number of cleaved Caspase3-positive cells was markedly lower in kidneys treated with JP4-039 and cisplatin compared to cisplatin-treated kidneys, regardless of whether JP4-039 was administered before or after cisplatin injection. This cytoprotective effect of JP4-039 on kidney tubular cells is consistent with the data from non-renal disease models in which JP4-039 administration reduced apoptotic cell death after gamma irradiation [24] or sodium sulfite [17] treatment. Our results also reveal that JP4-039 has potent anti-ferroptotic properties by preserving the cellular levels of GSH and the expression of *Gpx4* at baseline and significantly suppresses the expression of the pro-ferroptotic protein ACSL4 [34] in cisplatin-treated kidneys. The observed anti-ferroptotic activity of JP4-039 is in agreement with in vitro studies in which JP4-039 has been demonstrated to inhibit the activities of two different ferroptosis activators, i.e., erastin and RSL3 [14]. In fact, an analog of JP4-039, XJB-5-131, was recently shown to inhibit ferroptotic cell death in kidney ischemia reperfusion injury [35]. Intriguingly, our results indicate that there is at least a 24 h window after a low-dose cisplatin (10 mg/kg) administration in which kidney tubular cell function can be preserved by JP4-039 therapy in the kidney. This is highly significant as it closely correlates with the scenario in clinics, where interventions are needed after the occurrence of AKI. These results provide a foundation for future clinical studies to evaluate the efficacy of JP4-039 in preventing AKI in humans.

Together, our data demonstrate that JP4-039 protects the kidneys from cisplatin-induced AKI by inhibiting tubular oxidative stress and proinflammatory signaling and suppressing tubular cell death through apoptosis and ferroptosis pathways. Moreover, our studies show that JP4-039 can be administered both prophylactically and therapeutically to suppress AKI. Future studies will focus on assessing long term outcomes from JP4-039 treatment in additional kidney injury models, such as ischemia reperfusion injury.

## Data Availability

The original contributions presented in this study are included in the article/Supplementary Material. Further inquiries can be directed to the corresponding author(s).

## Supplementary Materials

The following supporting information can be downloaded at https://www.mdpi.com/article/10.3390/antiox13121534/s1: Table S1: List of antibodies and lectins used in the study; Table S2: List of quantitative RT-PCR primers.

## References

[1] Wang D., Lippard S.J. (2005). Cellular processing of platinum anticancer drugs. Nat. Rev. Drug Discov..

[2] Siddik Z.H. (2003). Cisplatin: Mode of cytotoxic action and molecular basis of resistance. Oncogene.

[3] Ozkok A., Edelstein C.L. (2014). Pathophysiology of cisplatin-induced acute kidney injury. Biomed. Res. Int..

[4] Kruidering M., Van de Water B., de Heer E., Mulder G.J., Nagelkerke J.F. (1997). Cisplatin-induced nephrotoxicity in porcine proximal tubular cells: Mitochondrial dysfunction by inhibition of complexes I to IV of the respiratory chain. J. Pharmacol. Exp. Ther..

[5] Forman H.J., Zhang H. (2021). Targeting oxidative stress in disease: Promise and limitations of antioxidant therapy. Nat. Rev. Drug Discov..

[6] Jiang M., Wei Q., Pabla N., Dong G., Wang C.Y., Yang T., Smith S.B., Dong Z. (2007). Effects of hydroxyl radical scavenging on cisplatin-induced p53 activation, tubular cell apoptosis and nephrotoxicity. Biochem. Pharmacol..

[7] Lieberthal W., Triaca V., Levine J. (1996). Mechanisms of death induced by cisplatin in proximal tubular epithelial cells: Apoptosis vs. necrosis. Am. J. Physiol..

[8] Wang J., Wang Y., Liu Y., Cai X., Huang X., Fu W., Wang L., Qiu L., Li J., Sun L. (2022). Ferroptosis, a new target for treatment of renal injury and fibrosis in a 5/6 nephrectomy-induced CKD rat model. Cell Death Discov..

[9] Ide S., Kobayashi Y., Ide K., Strausser S.A., Abe K., Herbek S., O’Brien L.L., Crowley S.D., Barisoni L., Tata A. (2021). Ferroptotic stress promotes the accumulation of pro-inflammatory proximal tubular cells in maladaptive renal repair. elife.

[10] Ide S., Ide K., Abe K., Kobayashi Y., Kitai H., McKey J., Strausser S.A., O’Brien L.L., Tata A., Tata P.R. (2022). Sex differences in resilience to ferroptosis underlie sexual dimorphism in kidney injury and repair. Cell Rep..

[11] Suzuki T., Motohashi H., Yamamoto M. (2013). Toward clinical application of the Keap1-Nrf2 pathway. Trends Pharmacol. Sci..

[12] Su L.J., Zhang J.H., Gomez H., Murugan R., Hong X., Xu D., Jiang F., Peng Z.Y. (2019). Reactive Oxygen Species-Induced Lipid Peroxidation in Apoptosis, Autophagy, and Ferroptosis. Oxidative Med. Cell. Longev..

[13] Friedmann Angeli J.P., Schneider M., Proneth B., Tyurina Y.Y., Tyurin V.A., Hammond V.J., Herbach N., Aichler M., Walch A., Eggenhofer E. (2014). Inactivation of the ferroptosis regulator Gpx4 triggers acute renal failure in mice. Nat. Cell Biol..

[14] Krainz T., Gaschler M.M., Lim C., Sacher J.R., Stockwell B.R., Wipf P. (2016). A Mitochondrial-Targeted Nitroxide Is a Potent Inhibitor of Ferroptosis. ACS Cent. Sci..

[15] Frantz M.C., Skoda E.M., Sacher J.R., Epperly M.W., Goff J.P., Greenberger J.S., Wipf P. (2013). Synthesis of analogs of the radiation mitigator JP4-039 and visualization of BODIPY derivatives in mitochondria. Org. Biomol. Chem..

[16] Shinde A., Berhane H., Rhieu B.H., Kalash R., Xu K., Goff J., Epperly M.W., Franicola D., Zhang X., Dixon T. (2016). Intraoral Mitochondrial-Targeted GS-Nitroxide, JP4-039, Radioprotects Normal Tissue in Tumor-Bearing Radiosensitive Fancd2(−/−) (C57BL/6) Mice. Radiat. Res..

[17] Glanzel N.M., Grings M., da Rosa-Junior N.T., de Carvalho L.M.C., Mohsen A.W., Wipf P., Wajner M., Vockley J., Leipnitz G. (2021). The mitochondrial-targeted reactive species scavenger JP4-039 prevents sulfite-induced alterations in antioxidant defenses, energy transfer, and cell death signaling in striatum of rats. J. Inherit. Metab. Dis..

[18] Airik M., Arbore H., Childs E., Huynh A.B., Phua Y.L., Chen C.W., Aird K., Bharathi S., Zhang B., Conlon P. (2023). Mitochondrial ROS Triggers KIN Pathogenesis in FAN1-Deficient Kidneys. Antioxidants.

[19] Berhane H., Shinde A., Kalash R., Xu K., Epperly M.W., Goff J., Franicola D., Zhang X., Dixon T., Shields D. (2014). Amelioration of radiation-induced oral cavity mucositis and distant bone marrow suppression in fanconi anemia Fancd2−/− (FVB/N) mice by intraoral GS-nitroxide JP4-039. Radiat. Res..

[20] Schneider C.A., Rasband W.S., Eliceiri K.W. (2012). NIH Image to ImageJ: 25 years of image analysis. Nat. Methods.

[21] Ruiz S., Pergola P.E., Zager R.A., Vaziri N.D. (2013). Targeting the transcription factor Nrf2 to ameliorate oxidative stress and inflammation in chronic kidney disease. Kidney Int..

[22] Shelton L.M., Lister A., Walsh J., Jenkins R.E., Wong M.H., Rowe C., Ricci E., Ressel L., Fang Y., Demougin P. (2015). Integrated transcriptomic and proteomic analyses uncover regulatory roles of Nrf2 in the kidney. Kidney Int..

[23] Pabla N., Dong Z. (2008). Cisplatin nephrotoxicity: Mechanisms and renoprotective strategies. Kidney Int..

[24] Thermozier S., Hou W., Zhang X., Shields D., Fisher R., Bayir H., Kagan V., Yu J., Liu B., Bahar I. (2020). Anti-Ferroptosis Drug Enhances Total-Body Irradiation Mitigation by Drugs that Block Apoptosis and Necroptosis. Radiat. Res..

[25] Li L., Guo C., Yu Y., Tie L., Lu G., Liu F., Han X., Ji L., Zou X. (2023). Differential effects of PGAM5 knockout on high fat high fructose diet and methionine choline-deficient diet induced non-alcoholic steatohepatitis (NASH) in mice. Cell Biosci..

[26] Lu Y., Sun Y., Liu Z., Lu Y., Zhu X., Lan B., Mi Z., Dang L., Li N., Zhan W. (2020). Activation of NRF2 ameliorates oxidative stress and cystogenesis in autosomal dominant polycystic kidney disease. Sci. Transl. Med..

[27] Shema R., Kulicke R., Cowley G.S., Stein R., Root D.E., Heiman M. (2015). Synthetic lethal screening in the mammalian central nervous system identifies Gpx6 as a modulator of Huntington’s disease. Proc. Natl. Acad. Sci. USA.

[28] Liu M., Reddy N.M., Higbee E.M., Potteti H.R., Noel S., Racusen L., Kensler T.W., Sporn M.B., Reddy S.P., Rabb H. (2014). The Nrf2 triterpenoid activator, CDDO-imidazolide, protects kidneys from ischemia-reperfusion injury in mice. Kidney Int..

[29] Wu J., Liu X., Fan J., Chen W., Wang J., Zeng Y., Feng X., Yu X., Yang X. (2014). Bardoxolone methyl (BARD) ameliorates aristolochic acid (AA)-induced acute kidney injury through Nrf2 pathway. Toxicology.

[30] Small D.M., Sanchez W.Y., Roy S.F., Morais C., Brooks H.L., Coombes J.S., Johnson D.W., Gobe G.C. (2018). N-acetyl-cysteine increases cellular dysfunction in progressive chronic kidney damage after acute kidney injury by dampening endogenous antioxidant responses. Am. J. Physiol. Renal. Physiol..

[31] Correia-Melo C., Marques F.D., Anderson R., Hewitt G., Hewitt R., Cole J., Carroll B.M., Miwa S., Birch J., Merz A. (2016). Mitochondria are required for pro-ageing features of the senescent phenotype. EMBO J..

[32] Ademowo O.S., Dias H.K.I., Burton D.G.A., Griffiths H.R. (2017). Lipid (per) oxidation in mitochondria: An emerging target in the ageing process?. Biogerontology.

[33] Nezu M., Souma T., Yu L., Suzuki T., Saigusa D., Ito S., Suzuki N., Yamamoto M. (2017). Transcription factor Nrf2 hyperactivation in early-phase renal ischemia-reperfusion injury prevents tubular damage progression. Kidney Int..

[34] Dai Y., Chen Y., Mo D., Jin R., Huang Y., Zhang L., Zhang C., Gao H., Yan Q. (2023). Inhibition of ACSL4 ameliorates tubular ferroptotic cell death and protects against fibrotic kidney disease. Commun. Biol..

[35] Zhao Z., Wu J., Xu H., Zhou C., Han B., Zhu H., Hu Z., Ma Z., Ming Z., Yao Y. (2020). XJB-5-131 inhibited ferroptosis in tubular epithelial cells after ischemia-reperfusion injury. Cell Death Dis..

