# Supplementary material for: JP4-039 Mitigates Cisplatin-Induced Acute Kidney Injury by Inhibiting Oxidative Stress and Blocking Apoptosis and Ferroptosis in Mice
Source: Antioxidants (Basel). 2024 Dec 15;13(12):1534. doi: 10.3390/antiox13121534 (PMC11727076; doi:10.3390/antiox13121534)
Supplement: Supplementary file 1 [file antioxidants-13-01534-s001.zip › antioxidants-3335707-supplementary.pdf]

Supplementary Table S1. Antibodies and lectins used in this study

**Antibody**	**Source**	**Identifier**
Rabbit 4-Hydroxynonenal (4-HNE)	R&D systems	MAB3249
Rabbit Collagen Type 1	Proteintech	14695-1-AP
Mouse Fibronectin	Proteintech	66042-1-Ig
Rat TIM-1/KIM-1/HAVCR	R&D systems	MAB1817
Rabbit Cleaved Caspase-3	Cell Signaling	#9661
LTL lectin	VectorLaboratories	FL-1321
Rabbit Anti-alpha Tubulin	Abcam	ab-15246
Rabbit Acsl4	Proteintech	22401-1-AP

Supplementary Table S2. qRT-PCR primers and NCBI gene accession numbers

Gene	Species	Forward	Reverse	NCBI gene accession numbers
Acsl4	mouse	TTGGCTACTTACCTTTGGCTC	AATCACCCTTGCTTCCCTTC	NM_019477.3
Ccl2	mouse	CCCAATGAGTAGGCTGGAGA	TCTGGACCCATTCCTTCTTG	NM_011333.3
Cxcl10	mouse	AAGTGCTGCCGTCATTTTCT	GTGGCAATGATCTCAACACG	NM_021274.2
Gapdh	mouse	GACTTCAACAGCAACTCCCA	TGTAGCCGTATTCATTGTCATACC	NM_008084.4
Gpx4	mouse	GCCTGGATAAGTACAGGGGTT	CATGCAGATCGACTAGCTGAG	NM_008162.4
Gpx6	mouse	GCCCAGAAGTTGTGGGGTTC	TCCATACTCATAGACGGTGCC	NM_145451.3
Havcr1	mouse	AGGAAGTCAGCATCTCTAAGCG	ACACAGAAAATCGCCTTGGC	NM_134248.2
Hmox	mouse	AAGCCGAGAATGCTGAGTTCA	GCCGTGTAGATATGGTACAAGGA	NM_010442.2
Il6	mouse	GTTCTCTGGGAAATCGTGGA	GGTACTCCAGAAGACCAGAGGA	NM_031168.2
Lcn2	mouse	CAAGCAATACTTCAAAATTACCCTGTA	GCAAAGCGGGTGAAACGTT	NM_008491.1
Nqo1	mouse	GGTAGCGGCTCCATGTACTC	CATCCTTCCAGGATCTGCAT	NM_008706.5
Nrf2	mouse	CAAGACTTGGGCCACTTAAAAGAC	AGTAAGGCTTTCCATCCTCATCAC	NM_010902.5
Tnf	mouse	GGAACTGGCAGAAGAGGCACTC	GCAGGAATGAGAAGAGGCTGAGAC	NM_013693.3
